# The genome sequence of the cinnamon sedge caddisfly, *Limnephilus marmoratus *(Curtis, 1834)

**DOI:** 10.12688/wellcomeopenres.18753.1

**Published:** 2023-02-08

**Authors:** Caleala Clifford, Kathy Friend, Sue Skipp, Ian Wallace, Natural History Museum Genome Acquisition Lab, Wellcome Sanger Institute Tree of Life programme, Wellcome Sanger Institute Scientific Operations: DNA Pipelines collective, Tree of Life Core Informatics collective, Benjamin W. Price, Darwin Tree of Life Consortium

**Affiliations:** 1 Natural Resources Wales, Cardiff, UK; 2 Environment Agency, London, UK; 3 British Caddis Recording Scheme, Wirral, UK; 4 Life Science Department, Natural History Museum, London, UK

**Keywords:** Limnephilus marmoratus, caddisfly, genome sequence, chromosomal, Trichoptera

## Abstract

We present a genome assembly from an individual *Limnephilus marmoratus* (a caddisfly; Arthropoda; Insecta; Trichoptera; Limnephilidae). The genome sequence is 1,630 megabases in span. Most of the assembly (99.93%) is scaffolded into 30 chromosomal pseudomolecules, including the assembled Z sex chromosome. The mitochondrial genome has also been assembled and is 15.4 kilobases in length.

## Species taxonomy

Eukaryota; Metazoa; Ecdysozoa; Arthropoda; Hexapoda; Insecta; Pterygota; Neoptera; Endopterygota; Trichoptera; Integripalpia; Plenitentoria; Limnephiloidea; Limnephilidae; Limnephilinae; Limnephilini; *Limnephilus*; *Limnephilus marmoratus* (Curtis, 1834) (NCBI:txid1271730).

## Background

**Figure 1.**
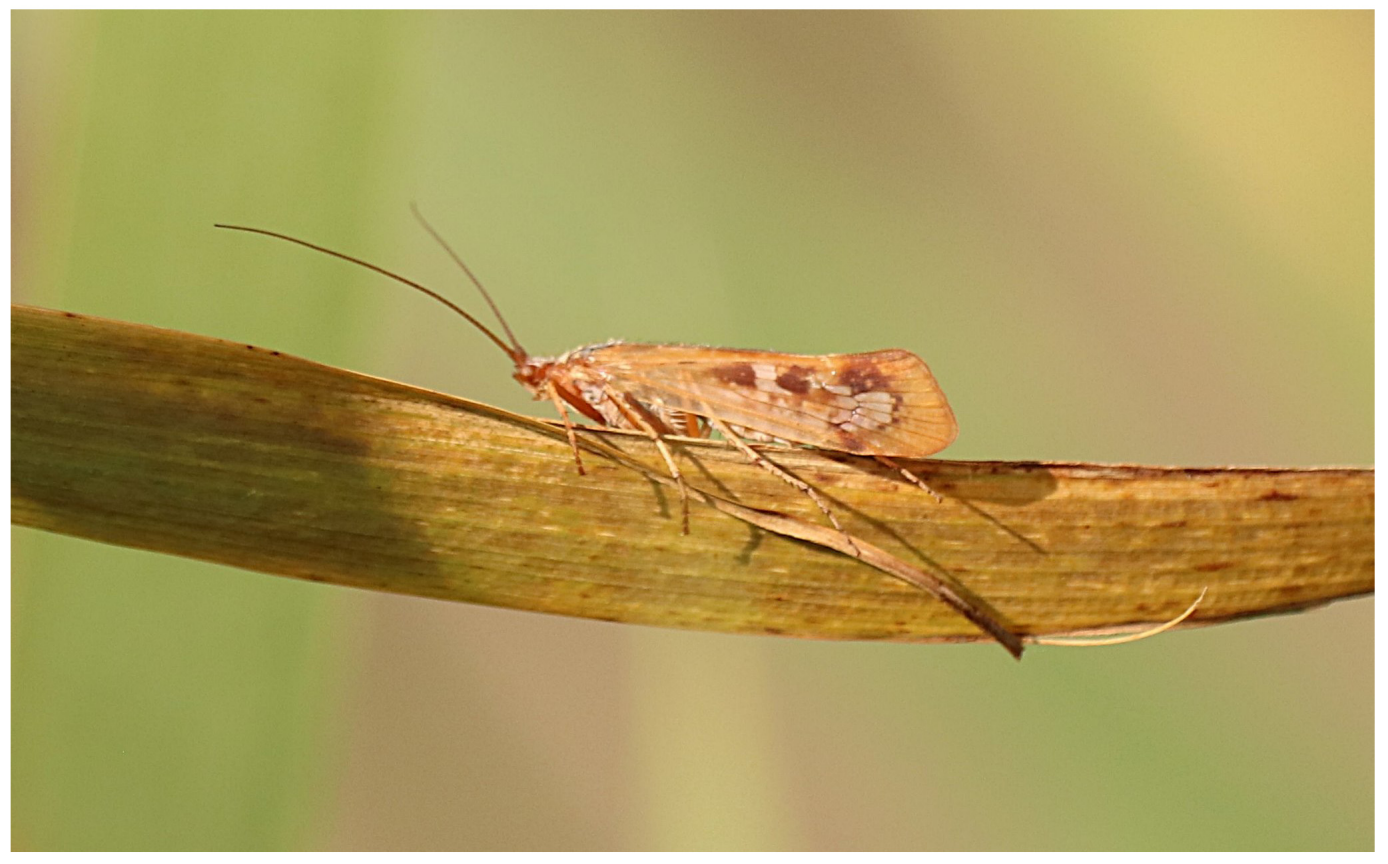
Photograph of *Limnephilus marmoratus* by jlaus (CC BY) on iNaturalist ( https://www.inaturalist.org/photos/96905588?size=original).

*Limnephilus marmoratus* ( Figure 1) is one of the most common British caddisflies, found from south-west England to Shetland, and is one of the caddis that share the common name of ‘cinnamon sedge’. Larvae can be found in still and slowly flowing waters of all sizes. Sites may dry up over summer, but this is a species more associated with waters that do not dry up completely. Larvae are found amongst submerged vegetation or debris such as twigs. The larvae feed primarily on dead plant litter, but can eat a wide range of food. The life cycle is not clear, as those from permanent waters seem to emerge as adults in summer and lay then, but the laying site is not known, whilst those from temporary waters emerge as adults in spring and diapause until laying in late summer on the damp bottom, e.g. under logs, where water will flood later that year. A similar life cycle that may or may not have a diapause is found in some other *Limnephilus* species.

The adult is one of the most variable of the larger caddis but always has wings with large patches of brown and white. It can be distinguished from its relatives using a key ( Barnard & Ross, 2012; Wallace *et al.*, 2022). The larvae are of a group that makes their case by arranging the cut pieces of vegetation at a tangent to the long axis of the case. The larvae can only be identified to species when dead or anaesthetised using a key by Wallace, Wallace and Philipson, 2003 which only works for final and penultimate instars; there is no key to identify small larvae, pupae or eggs.

The high-quality genome sequence described here is, to our knowledge, the first one reported for *L. marmoratus*, and has been generated as part of the Darwin Tree of Life project. It will aid in understanding the biology, physiology and ecology of the species, in addition to providing a mechanism to distinguish egg masses and early larval instars from those of its relatives.

## Genome sequence report

The genome was sequenced from a male *L. marmoratus* collected from Abbey Fields Lake Spring Source, UK (latitude 51.32, longitude 0.91). A total of 28-fold coverage in Pacific Biosciences single-molecule HiFi long reads and 55-fold coverage in 10X Genomics read clouds were generated. Primary assembly contigs were scaffolded with chromosome conformation Hi-C data. Manual assembly curation corrected 236 missing joins or misjoins and removed 48 haplotypic duplications, reducing the assembly length by 1.62% and the scaffold number by 65.82%, and increasing the scaffold N50 by 13.17%.

**Figure 2.**
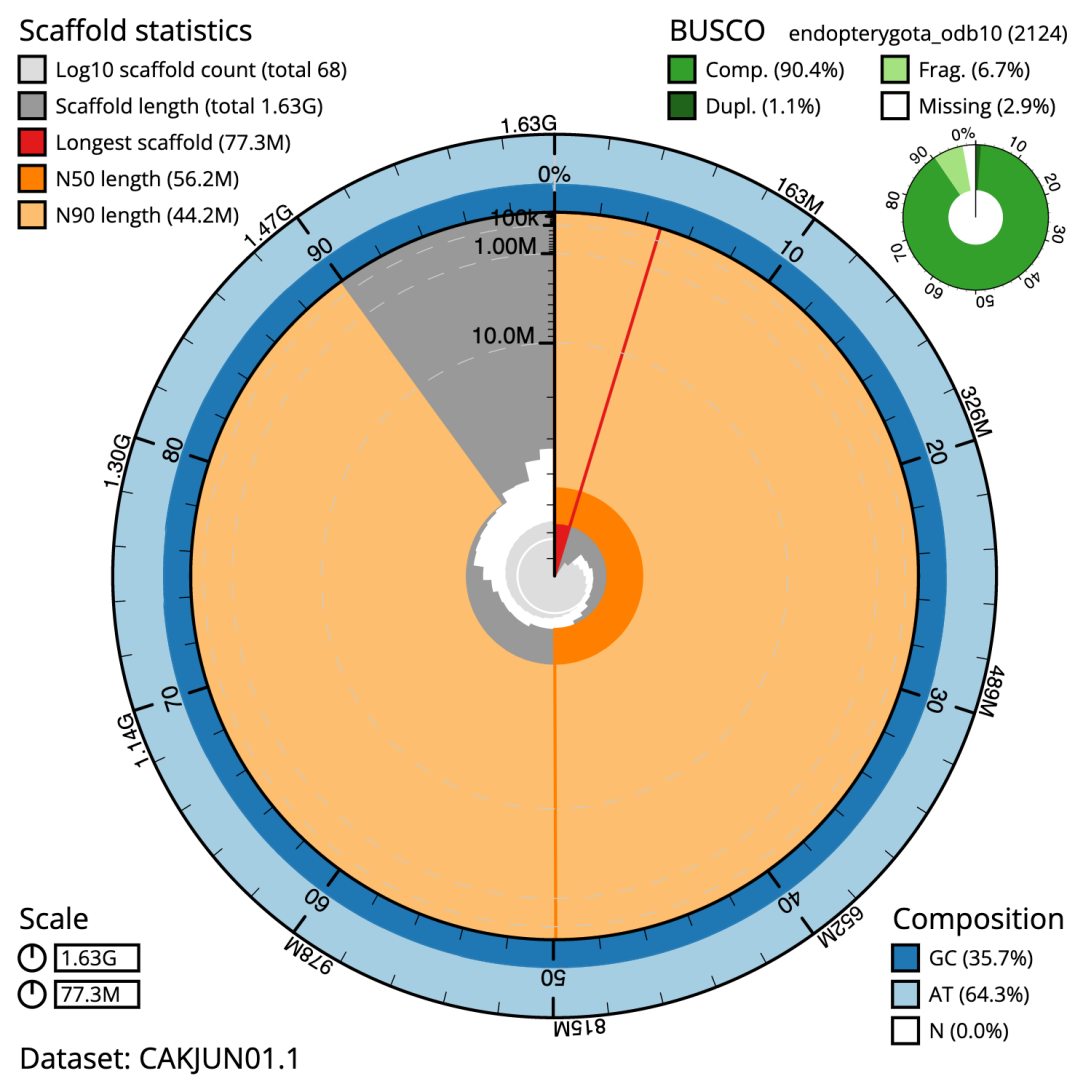
Genome assembly of *Limnephilus marmoratus*, iiLimMarm1.1: metrics. The BlobToolKit Snailplot shows N50 metrics and BUSCO gene completeness. The main plot is divided into 1,000 size-ordered bins around the circumference with each bin representing 0.1% of the 1,629,971,709 bp assembly. The distribution of chromosome lengths is shown in dark grey with the plot radius scaled to the longest chromosome present in the assembly (77,254,504 bp, shown in red). Orange and pale-orange arcs show the N50 and N90 chromosome lengths (56,174,236 and 44,182,302 bp), respectively. The pale grey spiral shows the cumulative chromosome count on a log scale with white scale lines showing successive orders of magnitude. The blue and pale-blue area around the outside of the plot shows the distribution of GC, AT and N percentages in the same bins as the inner plot. A summary of complete, fragmented, duplicated and missing BUSCO genes in the endopterygota_odb10 set is shown in the top right. An interactive version of this figure is available at https://blobtoolkit.genomehubs.org/view/iiLimMarm1.1/dataset/CAKJUN01.1/snail.

The final assembly has a total length of 1,630 Mb in 67 sequence scaffolds with a scaffold N50 of 56.2 Mb ( Table 1). Most (99.93%) of the assembly sequence was assigned to 30 chromosomal-level scaffolds, representing 29 autosomes and the Z sex chromosome ( Figure 2– Figure 5; Table 2). Chromosome-scale scaffolds confirmed by the Hi-C data are named in order of size. Chromosomes 2 and 8 have a high level of shared Hi-C signal which seems likely to result from a high proportion of shared repeat. While not fully phased, the assembly deposited is of one haplotype. Contigs corresponding to the second haplotype have also been deposited. The assembly has a BUSCO v5.3.2 ( Manni *et al.*, 2021) completeness of 90.4% using the endopterygota_odb10 reference set.

**Table 1.** Genome data for *Limnephilus marmoratus, iiLimMarm1.1*.

Project accession data	
Assembly identifier	iiLimMarm1.1
Species	*Limnephilus marmoratus*
Specimen	iiLimMarm1
NCBI taxonomy ID	1271730
BioProject	PRJEB46312
BioSample ID	SAMEA7520990
Isolate information	iiLimMarm1 (genome assembly and HiC), iiLimMarm3 (RNA)
Raw data accessions	
PacificBiosciences SEQUEL II	ERR6807999, ERR6939236
10X Genomics Illumina	ERR6688486–ERR6688494
Hi-C Illumina	ERR6688485, ERR6688490
PolyA RNA-Seq Illumina	ERR9434999
Genome assembly	
Assembly accession	GCA_917880885.1
Accession of alternate haplotype	GCA_917880875.1
Span (Mb)	1630.0
Number of contigs	394
Contig N50 length (Mb)	8.0
Number of scaffolds	67
Scaffold N50 length (Mb)	56.2
Longest scaffold (Mb)	77.3
BUSCO *	C:90.4%[S:89.3%,D:1.1%], F:6.7%,M:2.9%,n:2,124

*BUSCO scores based on the endopterygota_odb10 BUSCO set using v5.3.2. C = complete [S = single copy, D = duplicated], F = fragmented, M = missing, n = number of orthologues in comparison. A full set of BUSCO scores is available at https://blobtoolkit.genomehubs.org/view/iiLimMarm1.1/dataset/CAKJUN01.1/busco.

**Figure 3.**
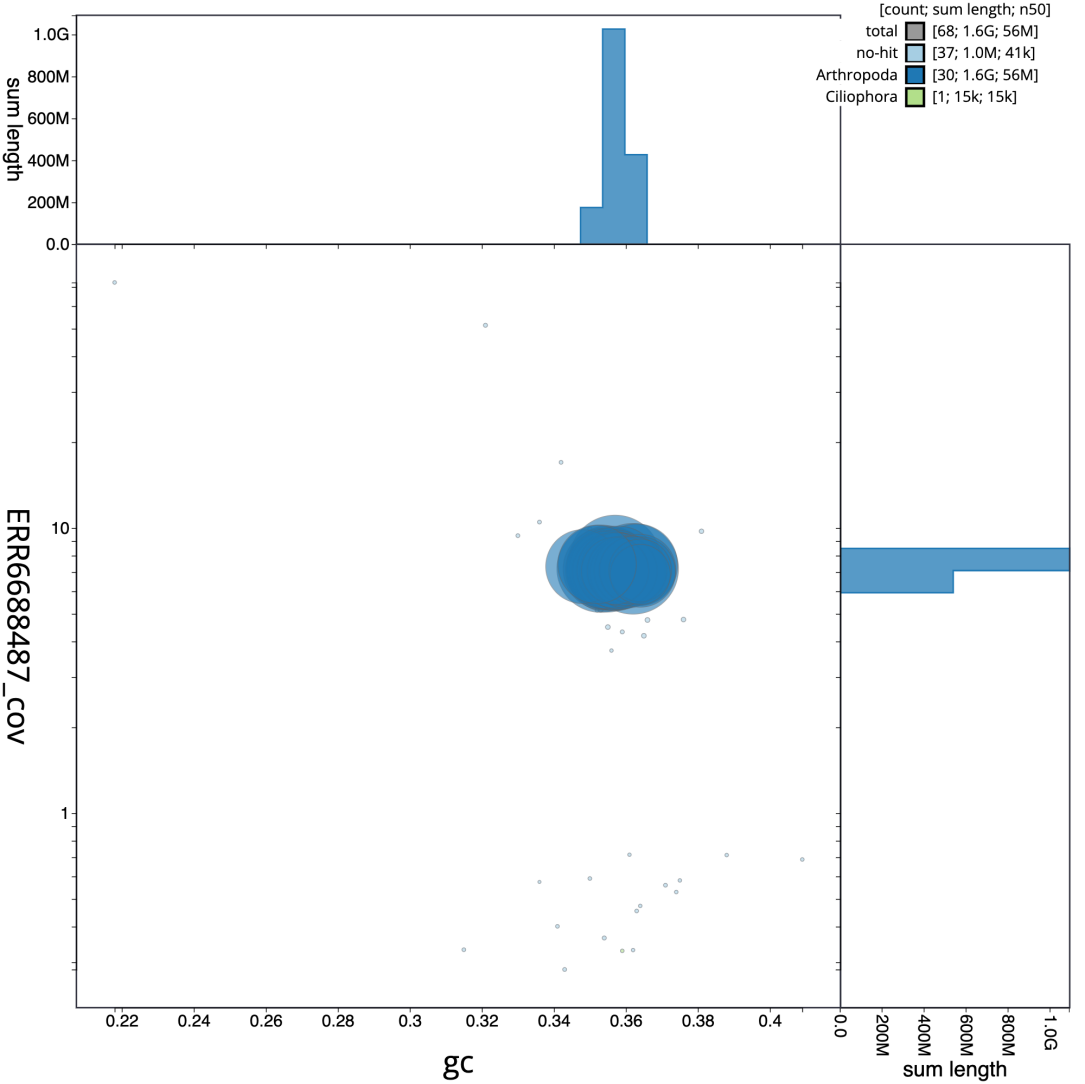
Genome assembly of *Limnephilus marmoratus*, iiLimMarm1.1: GC coverage. BlobToolKit GC-coverage plot. Scaffolds are coloured by phylum. Circles are sized in proportion to scaffold length. Histograms show the distribution of scaffold length sum along each axis. An interactive version of this figure is available at https://blobtoolkit.genomehubs.org/view/iiLimMarm1.1/dataset/CAKJUN01.1/blob.

**Figure 4.**
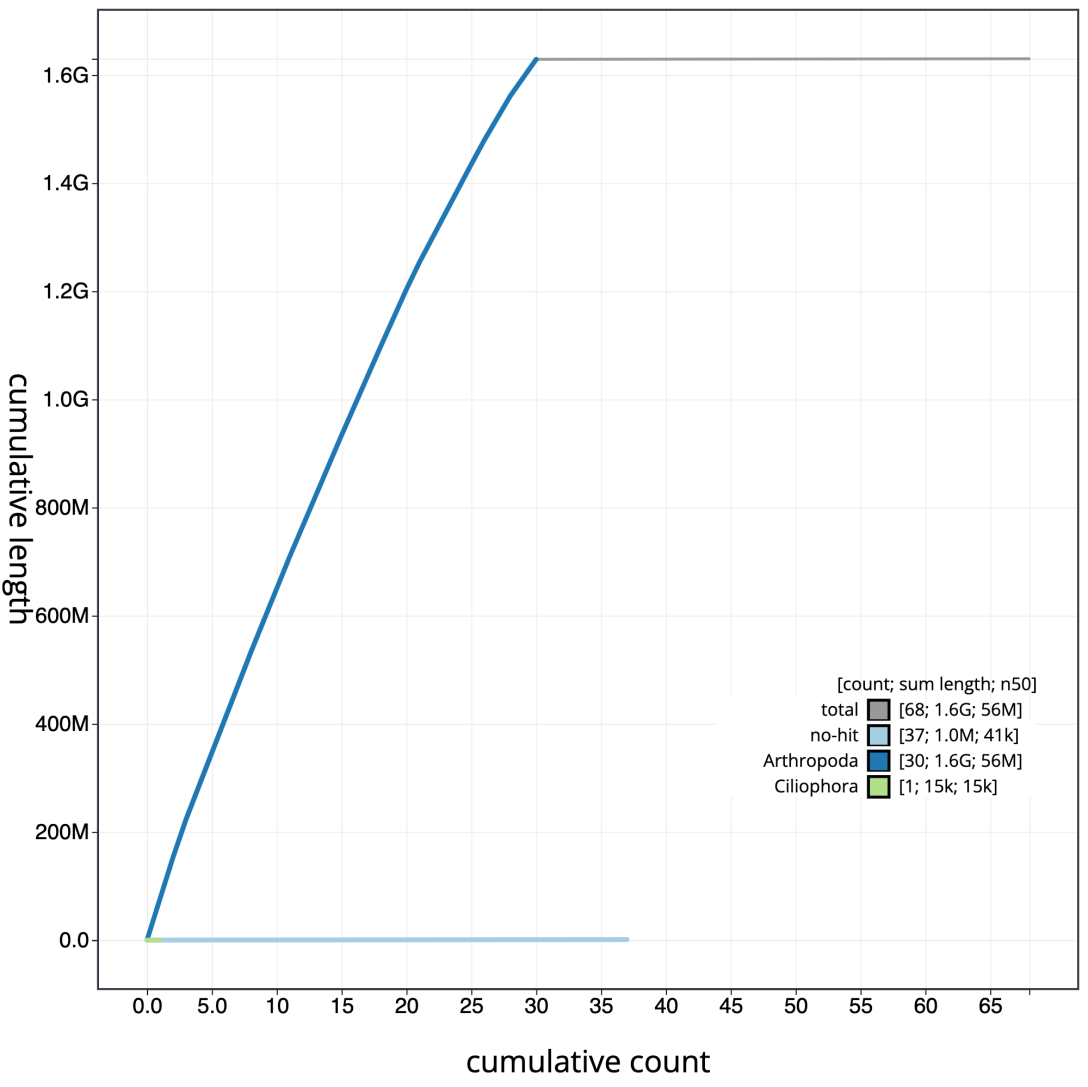
Genome assembly of *Limnephilus marmoratus*, iiLimMarm1.1: cumulative sequence. BlobToolKit cumulative sequence plot. The grey line shows cumulative length for all scaffolds. Coloured lines show cumulative lengths of scaffolds assigned to each phylum using the buscogenes taxrule. An interactive version of this figure is available at https://blobtoolkit.genomehubs.org/view/iiLimMarm1.1/dataset/CAKJUN01.1/cumulative.

**Figure 5.**
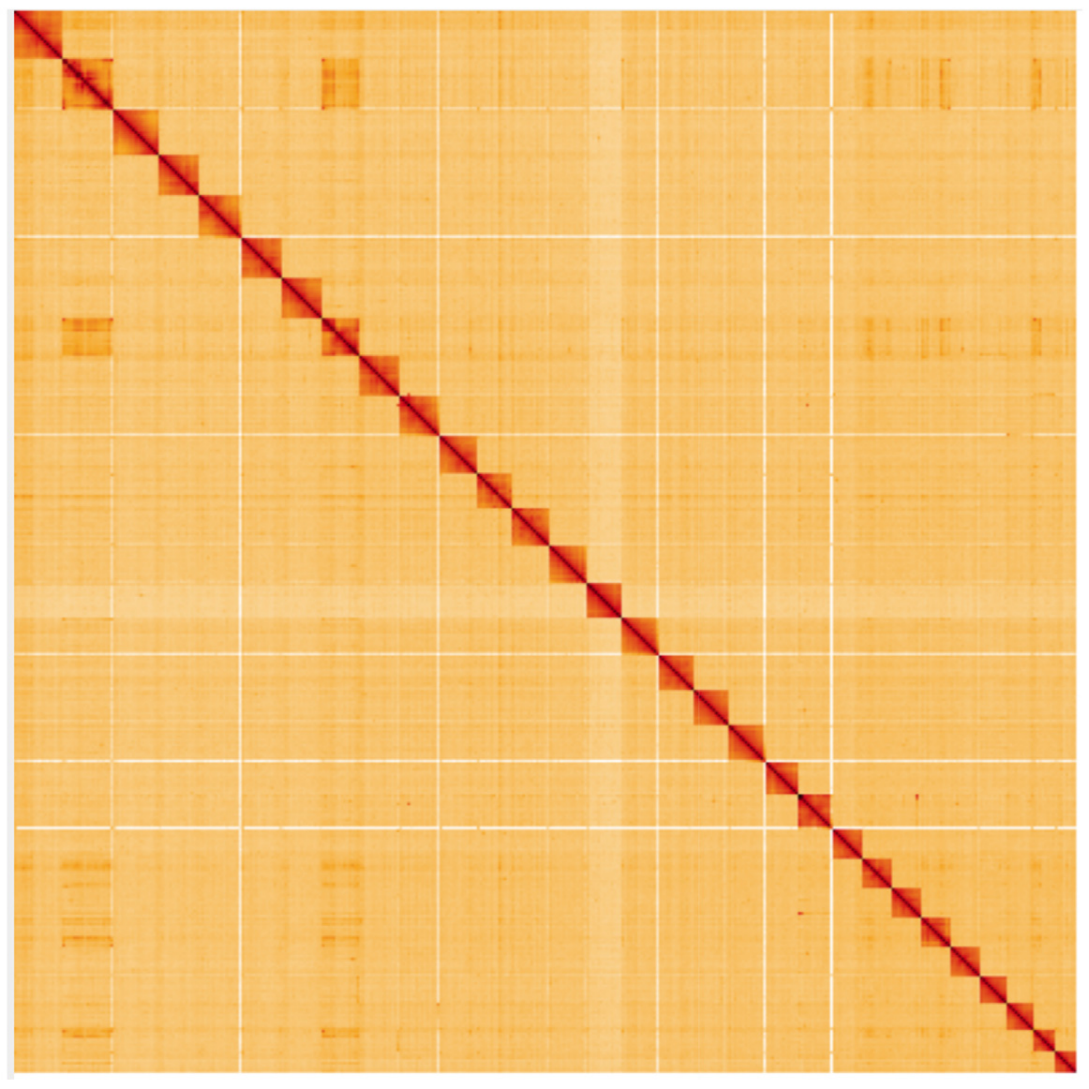
Genome assembly of *Limnephilus marmoratus*, iiLimMarm1.1: Hi-C contact map. Hi-C contact map of the iiLimMarm1.1 assembly, visualised using HiGlass. Chromosomes are shown in order of size from left to right and top to bottom. An interactive version of this figure may be viewed at https://genome-note-higlass.tol.sanger.ac.uk/l/?d=ZKeV_vHlTzyNCGL2cL5XEg.

**Table 2.** Chromosomal pseudomolecules in the genome assembly of *Limnephilus marmoratus*, iiLimMarm1.

INSDC accession	Chromosome	Size (Mb)	GC%
OU862906.1	1	77.25	35.7
OU862907.1	2	75.64	36.2
OU862908.1	3	69.9	35.3
OU862909.1	4	63.09	35.5
OU862910.1	5	62.56	35.5
OU862911.1	6	61.42	35.4
OU862912.1	7	61.26	35.6
OU862913.1	8	61.04	36.3
OU862914.1	9	60.58	35.5
OU862915.1	10	59	35.8
OU862916.1	11	57.72	35.6
OU862917.1	12	56.2	35.7
OU862918.1	13	56.17	35.7
OU862919.1	14	56.02	35.7
OU862921.1	15	54.71	35.5
OU862922.1	16	54.63	35.6
OU862923.1	17	54.38	35.4
OU862924.1	18	53.99	35.8
OU862925.1	19	52.31	35.7
OU862926.1	20	49.52	34.8
OU862927.1	21	46.13	36
OU862928.1	22	45.7	36.4
OU862929.1	23	45.28	36.1
OU862930.1	24	44.8	36.4
OU862931.1	25	44.18	35.9
OU862932.1	26	41.06	36.2
OU862933.1	27	40.83	35.7
OU862934.1	28	35.21	36.4
OU862935.1	29	32.33	36.4
OU862920.1	Z	55.98	35.2
OU862936.1	MT	0.02	21.8
-	unplaced	1.04	35.7

## Methods

### Sample acquisition and nucleic acid extraction

A male *Limnephilus marmoratus* (iiLimMarm1) was collected from Abbey Fields Lake Spring Source, UK (latitude 51.315207, longitude 0.9100518) by Kathy Friend and Sue Skipp (Environment Agency), and identified by Ian Wallace (British Caddis Recording Scheme). A second unsexed specimen (iiLimMarm3) was collected from Broadway Reen, Cardiff, Wales, UK (latitude 51.55, longitude –3.02) by Caleala Clifford (Natural Resources Wales). The specimens were taken from freshwater using a kick-net and snap-frozen in a dry shipper by Ben Price (Natural History Museum London).

DNA was extracted at the Tree of Life laboratory, Wellcome Sanger Institute. The iiLimMarm1 sample was weighed and dissected on dry ice with tissue set aside for Hi-C sequencing. Whole body tissue was cryogenically disrupted to a fine powder using a Covaris cryoPREP Automated Dry Pulveriser, receiving multiple impacts. High molecular weight (HMW) DNA was extracted using the Qiagen MagAttract HMW DNA extraction kit. Low molecular weight DNA was removed from a 20-ng aliquot of extracted DNA using 0.8X AMpure XP purification kit prior to 10X Chromium sequencing; a minimum of 50 ng DNA was submitted for 10X sequencing. HMW DNA was sheared into an average fragment size of 12–20 kb in a Megaruptor 3 system with speed setting 30. Sheared DNA was purified by solid-phase reversible immobilisation using AMPure PB beads with a 1.8X ratio of beads to sample to remove the shorter fragments and concentrate the DNA sample. The concentration of the sheared and purified DNA was assessed using a Nanodrop spectrophotometer and Qubit Fluorometer and Qubit dsDNA High Sensitivity Assay kit. Fragment size distribution was evaluated by running the sample on the FemtoPulse system.

RNA was extracted from whole body tissue of iiLimMarm3 in the Tree of Life Laboratory at the WSI using TRIzol, according to the manufacturer’s instructions. RNA was eluted in 50 μL RNAse-free water and its concentration assessed using a Nanodrop spectrophotometer and Qubit Fluorometer using the Qubit RNA Broad-Range (BR) Assay kit. Analysis of the integrity of the RNA was done using Agilent RNA 6000 Pico Kit and Eukaryotic Total RNA assay.

### Sequencing

Pacific Biosciences HiFi circular consensus and 10X Genomics read cloud DNA sequencing libraries were constructed according to the manufacturers’ instructions. Poly(A) RNA-Seq libraries were constructed using the NEB Ultra II RNA Library Prep kit. DNA and RNA sequencing was performed by the Scientific Operations core at the WSI on Pacific Biosciences SEQUEL II (HiFi), Illumina NovaSeq 6000 (RNA-Seq) and HiSeq X Ten (10X) instruments. Hi-C data were also generated from whole body tissue of iiLimMarm1 and iiLimMarm3 using the Arimav2 kit and sequenced on the Illumina NovaSeq 6000 instrument.

### Genome assembly

Assembly was carried out with Hifiasm ( Cheng *et al.*, 2021) and haplotypic duplication was identified and removed with purge_dups ( Guan *et al.*, 2020). One round of polishing was performed by aligning 10X Genomics read data to the assembly with Long Ranger ALIGN, calling variants with freebayes ( Garrison & Marth, 2012). The assembly was then scaffolded with Hi-C data ( Rao *et al.*, 2014) using SALSA2 ( Ghurye *et al.*, 2019). The assembly was checked for contamination as described previously ( Howe *et al.*, 2021). Manual curation was performed using HiGlass ( Kerpedjiev *et al.*, 2018) and Pretext ( Harry, 2022). The mitochondrial genome was assembled using MitoHiFi ( Uliano-Silva *et al.*, 2021), which performed annotation using MitoFinder ( Allio *et al.*, 2020). The genome was analysed and BUSCO scores generated within the BlobToolKit environment ( Challis *et al.*, 2020). Table 3 contains a list of all software tool versions used, where appropriate.

**Table 3.** Software tools and versions used.

Software tool	Version	Source
BlobToolKit	3.4.0	Challis *et al*., 2020
freebayes	1.3.1-17- gaa2ace8	Garrison & Marth, 2012
Hifiasm	0.15.3	Cheng *et al*., 2021
HIGlass	1.11.6	Kerpedjiev *et al*., 2018
Long Ranger ALIGN	2.2.2	https://support.10xgenomics.com/ genome-exome/software/pipelines/ latest/advanced/other-pipelines
MitoHiFi	2.0	Uliano-Silva *et al*., 2021
PretextView	0.2	Harry, 2022
purge_dups	1.2.3	Guan *et al*., 2020
SALSA	2.2	Ghurye *et al*., 2019

### Ethics/compliance issues

The materials that have contributed to this genome note have been supplied by a Darwin Tree of Life Partner. The submission of materials by a Darwin Tree of Life Partner is subject to the Darwin Tree of Life Project Sampling Code of Practice. By agreeing with and signing up to the Sampling Code of Practice, the Darwin Tree of Life Partner agrees they will meet the legal and ethical requirements and standards set out within this document in respect of all samples acquired for, and supplied to, the Darwin Tree of Life Project. Each transfer of samples is further undertaken according to a Research Collaboration Agreement or Material Transfer Agreement entered into by the Darwin Tree of Life Partner, Genome Research Limited (operating as the Wellcome Sanger Institute), and in some circumstances other Darwin Tree of Life collaborators.

## Data Availability

European Nucleotide Archive: *Limnephilus marmoratus*. Accession number PRJEB46312; https://identifiers.org/ena.embl/PRJEB46312 ( Wellcome Sanger Institute, 2022) The genome sequence is released openly for reuse. The *Limnephilus marmoratus* genome sequencing initiative is part of the Darwin Tree of Life (DToL) project. All raw sequence data and the assembly have been deposited in INSDC databases. The genome will be annotated using available RNA-Seq data and presented through the Ensembl pipeline at the European Bioinformatics Institute. Raw data and assembly accession identifiers are reported in Table 1.

## Author information

Members of the Natural History Museum Genome Acquisition Lab are listed here: https://doi.org/10.5281/zenodo.4790043.

Members of the Wellcome Sanger Institute Tree of Life programme are listed here: https://doi.org/10.5281/zenodo.4783585.

Members of Wellcome Sanger Institute Scientific Operations: DNA Pipelines collective are listed here: https://doi.org/10.5281/zenodo.4790455.

Members of the Tree of Life Core Informatics collective are listed here: https://doi.org/10.5281/zenodo.5013541.

Members of the Darwin Tree of Life Consortium are listed here: https://doi.org/10.5281/zenodo.4783558.
