# Supplementary material for: The genome sequence of the cinnamon sedge caddisfly, Limnephilus marmoratus (Curtis, 1834)
Source: Wellcome Open Res. 2023 Feb 8;8:64. [Version 1] doi: 10.12688/wellcomeopenres.18753.1 (PMC10509604; doi:10.12688/wellcomeopenres.18753.1)
Supplement: Peer review history

# Reviewer report: Reviewer response for version 1

**DOI:** 10.21956/wellcomeopenres.20793.r65704

**Published:** 2023-09-19

**Authors:** Xi-Ling Deng

**Affiliations:** 1 Senckenberg Research Institute and Natural History Museum, Senckenberg, Germany

*Limnephilus *is a group of case-maker caddisflies. It plays an important role in freshwater ecosystems. This article reported a chromosome-level genome of a *Limnephilus *species. This genome is of high quality. The data was well processed and the results were nicely visualized.

 In line with another reviewer's comment in the genome note of *Glyphotaelius pellucidus*, it would be good to make a comparison with other caddisfly genomes generated by Darwin Tree of Life. It would also be very helpful if the author could add some additional information about the challenges they have faced in the lab, such as DNA extraction, because sometimes it is difficult to get good-quality DNA of caddisflies.

Are sufficient details of methods and materials provided to allow replication by others?

Yes

Is the rationale for creating the dataset(s) clearly described?

Yes

Are the datasets clearly presented in a useable and accessible format?

Yes

Are the protocols appropriate and is the work technically sound?

Yes

Reviewer Expertise:

Population genomics

I confirm that I have read this submission and believe that I have an appropriate level of expertise to confirm that it is of an acceptable scientific standard.

# Reviewer report: Reviewer response for version 1

**DOI:** 10.21956/wellcomeopenres.20793.r54827

**Published:** 2023-02-10

**Authors:** Jacqueline Heckenhauer

**Affiliations:** 1 LOEWE Centre for Translational Biodiversity Genomics (LOEWE-TBG), Frankfurt, Germany

In their data note, the authors present ****the genome sequence of the cinnamon sedge caddisfly, *Limnephilus marmoratus *using a *de novo* assembly method following the approach used by the Darwin Tree of Life Project. The presented genome is of high quality and released openly for reuse. To my knowledge this is the first time the genome of this species has been assembled, therefore this high-quality reference genome is very beneficial to the field and I approve the article.

 However, I have some comments/suggestions:

1. Methods: No details are given with respect to the parameters of the bioinformatic tools used. Maybe it is possible to add the command used for assembly and quality control or refer to a data note which already includes this exact information since I imagine the pipeline for genome assembly is similar to other genomes assembled in the DToL Project. 
2. Other quality metrics besides BUSCO and N50 would be interesting to report in addition (for example assessing assembly continuity using LTR-RTs, and reporting back mapping rate of reads to the assembly...)
3. Was the assembly length as long as the expected genome size?
4. I know that there is an alternate assembly for this specimen. This is really cool! I see the accession of the alternate haplotype is mentioned in the table but maybe also mention this in the text?

Are sufficient details of methods and materials provided to allow replication by others?

Partly

Is the rationale for creating the dataset(s) clearly described?

Yes

Are the datasets clearly presented in a useable and accessible format?

Yes

Are the protocols appropriate and is the work technically sound?

Yes

Reviewer Expertise:

Biodiversity genomics, aquatic insects, phylogenetics, comparative genomics

I confirm that I have read this submission and believe that I have an appropriate level of expertise to confirm that it is of an acceptable scientific standard.
